# Clinical utility of metagenomic next-generation sequencing (mNGS) and a novel PCR-based point-of-care testing (POCT) for pathogen detection in pulmonary infections: a retrospective study

**DOI:** 10.1186/s12879-025-11814-5

**Published:** 2025-11-26

**Authors:** Ao Shen, Xiaoming Xu, Lei Xu, Xifang Nie, Jingwen Ai, Weijun Chen

**Affiliations:** 1 College of Life Sciences, University of Chinese Academy of Sciences, Beijing, 100049 China; 2 BGI Genomics, Shenzhen, 518083 China; 3 Clin Lab, BGI Genomics, Tianjin, 300308 China; 4 Department of Infectious Disease, Huashan Hospital of Fudan University, Shanghai, 200040 China

**Keywords:** Point-of-caretesting (POCT), Metagenomic next-generation sequencing (mNGS), Pulmonary infection, Mixed infection, Pathogen

## Abstract

**Background:**

Metagenomic next-generation sequencing (mNGS) and multiple point-of-care testing (POCT) techniques have demonstrated significant potential in pathogen detection. However, neither technology fully meets all clinical diagnostic needs for pulmonary infections. This study aimed to evaluate the complementary detection performance and clinical applicability of POCT and mNGS in pulmonary infections, using conventional culture as a reference.

**Methods:**

This study enrolled forty-five patients with suspected lower respiratory tract infections for concurrent evaluation using POCT and mNGS. The detection performance of traditional culture methods, POCT, and mNGS was subsequently analyzed and compared.

**Results:**

Both molecular methods showed high positive detection rates, surpassing that of the culture method. When conventional culture was used as the gold standard, the sensitivity and positive predictive value (PPV) within the detection range of the PM Easy Lab Respiratory Panel (RP) were 97.1% and 80.5%, respectively, whereas for mNGS, these values were 90.2% and 92.5%, respectively. A comparison of the PM Easy Lab RP and mNGS results revealed that the PM Easy Lab was faster (100 min vs. 24 h) and more sensitive (88 vs. 63 pathogens) within its detection range, whereas mNGS offered a broader spectrum of pathogen detection. The overall consistency between PM Easy Lab RP and mNGS was 88.9%. *Klebsiella pneumoniae* and *Acinetobacter baumannii* were identified as the most prevalent bacterial infections by all three detection methods. Moreover, both PM Easy Lab RP and mNGS demonstrated enhanced capability over culture in detecting mixed infections (57.8%, 84.4% vs. 15.6%, all *P* < 0.01), uncovering a substantial number of viral and bacterial-viral co-infections that are undetectable by conventional culture methods. The most common combination of mixed infections in the PM Easy Lab RP was mixed bacterial infections (76.9%, 20/26), whereas in mNGS, it was bacterial-fungal-viral mixed infections (36.8%, 14/38).

**Conclusions:**

The findings indicate that PM Easy Lab RP and mNGS offer distinct yet complementary value. PM Easy Lab has potential advantages in speed, sensitivity, and efficiency in detecting pathogens within its panel, and it could be considered for rapid, frontline testing, while mNGS provides a broad-spectrum detection capability, making it more suitable for comprehensive investigation of complex cases, though careful clinical interpretation is required to distinguish pathogenic from colonizing or contaminating organisms. Understanding their respective strengths can guide the development of optimized, hierarchical diagnostic pathways.

**Supplementary Information:**

The online version contains supplementary material available at 10.1186/s12879-025-11814-5.

## Introduction

Infectious diseases continue to pose a significant global public health challenge, with lower respiratory tract infections being a primary cause of high morbidity and mortality [1, 2]. These infections can be caused by various pathogens, including bacteria, fungi, viruses, and parasites. Rapid and accurate identification of causative agents is crucial for effective diagnosis and for minimizing the overuse of broad-spectrum antibiotics. However, the current gold standard for clinical microbiological detection, culture-based methods, has limitations. These methods are time-consuming and primarily effective only for bacterial and fungal tests, with low positive detection rates, particularly for fastidious organisms [3, 4]. Given the diversity of clinically relevant infectious pathogens, traditional culture methods often fall short of meeting clinical needs. Consequently, there is an urgent requirement for novel diagnostic methods that offer higher sensitivity, broader spectrum detection, and faster results for clinical applications.

With recent advancements in molecular biology, numerous culture-independent techniques have been applied for pathogen detection, increasing sensitivity and accuracy. Metagenomic next-generation sequencing (mNGS), a hypothesis-independent method, enables simultaneous detection of bacteria, fungi, viruses, and parasites in a single test and is capable of identifying novel and unknown pathogens such as SARS-CoV-2 [5]. mNGS technology has been extensively utilized for clinical diagnosis of various infectious diseases, including bloodstream infections [6], central nervous system infections [7], respiratory tract infections [3], urinary tract infections [8], and prosthetic joint infections [9]. Despite demonstrating good sensitivity and specificity in clinical applications, mNGS has not been widely implemented in clinical laboratories due to its requirement for sophisticated experimental conditions, complex protocols, and lengthy turnaround times. Conversely, Point-of-care testing (POCT) is a diagnostic approach that enables rapid, on-site results through various promising technologies. It transforms traditional nucleic acid detection methods into integrated, closed, simplified, and automated processes, allowing detection to occur near or at the patient’s location. Previous studies have demonstrated that compared with traditional laboratory tests, POCT offers significant advantages in reducing patient treatment costs and hospital stays while providing faster results [10, 11]. The PM Easy Lab Respiratory Panel (RP) (BGI PathoGenesis, Shenzhen, China) is a sample-to-answer PCR-based test that analyzes respiratory tract samples for the presence of bacteria and viruses within approximately 100 min, requiring less than 5 min of hands-on time. Several similar platforms, including the ePlex RP (GenMark Diagnostics) and the BioFire FilmArray Respiratory 2 (RP2) panels (BioFire Diagnostics), have been fully validated for clinical use, with comparative studies reported [12, 13]. These studies have shown that POCTs are user-friendly in clinical practice and exhibit good detection sensitivity. However, performance comparisons between mNGS and PCR-based POCTs remain rare.

The primary objective of this study was to compare the clinical utility and performance characteristics of mNGS and a novel PCR-based POCT method (PM Easy Lab RP) for detecting pathogens in patients with pulmonary infections, with conventional culture serving as a reference method for cultivable organisms. Rather than seeking to declare a superior technology, this study was designed to simultaneously apply these complementary diagnostic methods to a single cohort of pulmonary infections. We aimed to elucidate their potential complementary and hierarchical roles in a modern diagnostic workflow, thereby providing data to inform the rational deployment of these tools based on clinical scenario and resource availability.

## Methods

### Sample selection

We performed a retrospective cohort study of 45 residual samples suspected of having complex lower respiratory tract infections that were sent to Tianjin Medical Laboratory of BGI for mNGS testing between November 2019 and April 2022. While a uniform set of clinical criteria was not prospectively applied, the documented clinical symptoms and the fact that samples were sent for microbiological testing confirm the presence of a clinical suspicion of infection. We retrospectively collected the clinical records attached to the samples when they were sent for inspection and selected the samples containing clinical culture information and with a remaining sample volume of more than 1 ml for inclusion in this study. Data on demographic characteristics, clinical symptoms, underlying diseases, and clinical treatment were all obtained from clinical records. Before testing, all the samples were anonymously processed and stored at − 80 °C.

### Conventional culture and antimicrobial susceptibility testing

Due to the retrospective nature of this study and the multi-center origin of samples, the specific methodologies and platforms used for conventional culture and antimicrobial susceptibility testing (AST) varied across the contributing hospitals and were not available for detailed reporting. The culture and AST results utilized for comparison in this analysis were those formally reported in the patients’ clinical records, generated according to the standard operating procedures of each hospital’s clinical microbiology laboratory, which are consistent with guidelines from the Chinese Committee for Clinical Laboratory Standards (CCCLS) and international standards. In this study, four samples were classified as “indeterminate” or “negative” due to positive culture results in the absence of species-level identification.

### PM easy lab respiratory panel (RP) testing

Approximately 400 µl of the original bronchoalveolar lavage fluid (BALF) sample or preliquefied sputum sample was subjected to PM Easy Lab RP testing. This is a sample-to-answer, multiplex qPCR-based POCT system designed to simultaneously detect 18 common respiratory pathogens.

The PM Easy Lab RP test consists of automated nucleic acid extraction, reverse transcription, nucleic acid amplification, and result analysis in 100 minutes per run. One sample was processed at a time, and the manual operation was less than 5 minutes for each run procedure. The analysis software performs automated result analysis, with each target in a valid run reported as ‘detected’ or ‘not detected’, whereas the internal controls are reported as ‘Pass’ or ‘Fail’. If any of the internal controls reported failed, the test results for this batch were considered invalid. The final report is presented on the all-in-one display without the need for additional PC equipment.

There were 18 targets, including 9 bacteria, 2 DNA viruses, and 7 RNA viruses. The detailed species information and corresponding limits of detection are shown in Table 1.

**Table 1:** Analytes detected by the PM easy lab RP

Analyte	Limit of detection (LoD)
DNA Viruses	
Adenovirus	500copies/mL
Bocavirus	500copies/mL
RNA Viruses	
Coronavirus	500copies/mL
Influenza A virus	500copies/mL
Influenza B virus	500copies/mL
Rhinovirus	500copies/mL
Respiratory syncytial virus	500copies/mL
Parainfluenza virus	500copies/mL
Bacteria	
*Acinetobacter baumannii*	300copies/mL
Chlamydia pneumoniae	1000copies/mL
Haemophilus influenza	1000copies/mL
*Klebsiella pneumoniae*	500copies/mL
*Legionella pneumophila*	500copies/mL
Mycoplasma pneumonia	1000copies/mL
*Pseudomonas aeruginosa*	500copies/mL
*Staphylococcus aureus*	500copies/mL
*Streptococcus pneumoniae*	500copies/mL

The LoD values were provided by the manufacturer based on analytical validation studies

### Metagenomic next-generation sequencing

BALF and preliquefied sputum samples were subjected to a host depletion procedure to remove host nucleic acid before DNA extraction. The clinical samples were mixed with saponin to destroy the human cell membrane specifically, followed by Deoxyribonuclease (Vazyme Biotech, Nanjing, China) treatment to remove free nonencapsulated RNA and DNA from the sample, leaving intact microorganisms for downstream extraction. The detailed procedures can refer to the previous literature by Hasan et al. [14].

For DNA extraction, before extraction, 500 µl samples were mixed with 7.2 µl of lyticase (RT410, Tiangen Biotech, Beijing, China) and incubated at 30 °C for 10 min. After a short centrifugation, all the samples were transferred to a FastPrep (FP) Lysis tube that included 250 µl of 0.5 mm glass beads. Then, the samples were processed by bead beating twice at 6 m/s for 45 s each on a FastPrep-24 bead beater (MP Biomedicals, Santa Ana, CA, USA), with a 2-minute incubation between bead-beating cycles. The homogenized sample was centrifuged at 2000 rpm for 20 s, and 300 µl of the supernatant was transferred to a 1.5 mL extraction tube. Two microliters of internal DNA was added to the supernatant, and then DNA was extracted via the Magnetic Pathogenic Microorganism DNA/RNA Kit (NG550, Tiangen Biotech, Beijing, China) according to the manufacturer’s instructions. For RNA extraction, 500 µL samples were transferred to a 1.5 mL tube, 2 µL of RNA internal standard, and proceeded directly with Magnetic Pathogenic Microorganism DNA/RNA Kit (NG550, Tiangen Biotech, Beijing, China), following the instructions provided in the kit.

A single-stranded DNA circle (ssDNA circle) library was constructed after reverse transcription (only RNA), DNA fragmentation, end repair, adapter ligation, PCR amplification, DNA denaturation into single strands, and DNA circularization. DNA nanoballs (DNBs) were generated from circular ssDNA by rolling circle amplification (RCA). Finally, the qualified DNBs were loaded on the flow cell and sequenced (50 bp, single end) on the PMSEQ-4500 platform (MGI, Shenzhen, China), and an average of 40 million reads were obtained for each sample.

The raw data were preprocessed by removing adapter reads, low-quality reads, and short reads via in-house software, and then the human reads and internal reference reads were calculated using Burrows‒Wheeler alignment (BWA, version: 0.7.17-r1188). Subsequently, the remaining data were identified through BWA alignment to the self-built pathogen sequence database, which included approximately 17,500 microorganism reference sequences of bacteria, fungi, viruses, and parasites. Finally, pathogens were annotated with all classified mapped reads. The strictly mapped read number (SMRN) for each pathogen was defined as the number of reads showing > 95% sequence identity to pathogen reference sequences after alignment. This stringent threshold was applied to minimize false-positive alignments and ensure high-confidence pathogen identification. The basic criteria for the thresholds of the mNGS results were as follows: (I) the SMRN of microorganisms in a sample could be distinguished from that of negative samples; (II) the SMRN of DNA pathogens with bacteria, viruses, fungi, mycoplasma and chlamydia was not less than 3; for RNA viruses, the SMRN was not less than 1; (III) for parasites, the SMRN was not less than 100; and (IV) for the *Mycobacterium tuberculosis* complex and other intracellular bacteria, such as Brucella, *Legionella pneumophila* and Nocardia, the SMRN was not less than 1.

### Statistical analysis

The calculations of medians, interquartile ranges (IQRs), and 95% confidence intervals (CIs) were performed with IBM SPSS 25.0 (IBM Corp., Armonk, NY, USA) and GraphPad Prism 8.0.1 (GraphPad Software, San Diego, CA, USA). The sensitivity, specificity, positive predictive value (PPV), and negative predictive value (NPV) were calculated through the established 2 × 2 contingency tables. Pearson’s chi-square (χ^2^) test or Fisher’s exact test was used to compare the analysis of data from different methods. A *P* value of < 0.05 was considered to indicate statistical significance.

## Results

### Participant demographic information

A total of 45 patients (31 male and 14 female) diagnosed with pulmonary infections between November 2019 and April 2022 were included. The patients ranged in age from 1 to 91 years, with an average age of 59.89 ± 26.31 years. Among the 45 patients, 13 (28.9%) were diagnosed with severe pneumonia, and 17 (37.8%) had diverse medical comorbidities, including bronchiectasis, diabetes, hypertension, coronary artery disease, cholecystitis, acute renal failure, autoimmune disease, interstitial lung disease, chronic liver disease, solid-organ transplantation, and leukemia. The most common clinical manifestations included fever (57.8%), cough (42.2%), dyspnea (8.9%), and chest pain/chest tightness (8.9%). Among these patients, 37 also had inflammatory indicators, such as C-reactive protein (CRP) and procalcitonin (PCT), which reflect the state of the body after infection to varying degrees, and the average CRP and PCT levels were 43.5 and 2.6, respectively. The detailed characteristics of the enrolled patients are shown in Table S1.

### Pathogen profiles detected by conventional culture methods

**Fig. 1:**
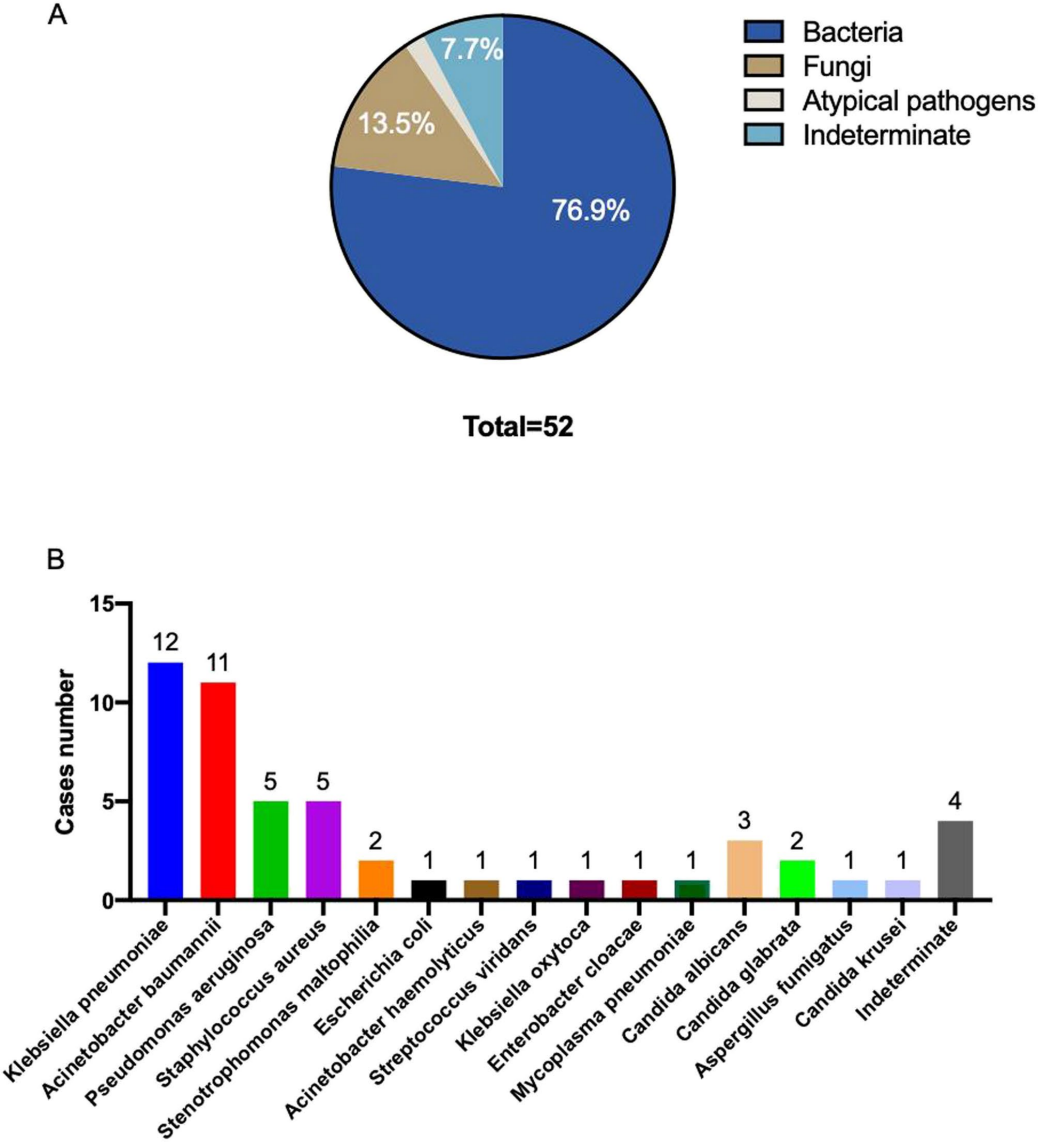
Distribution of pathogens identified by culture. **A** Percentage of various pathogens detected by culture. “Indeterminate” refers to samples with positive culture results that lacked species-level identification. **B** Pathogen species and the corresponding number of cases identified by culture

A total of 45 patients were enrolled, and detailed clinical records were used for analysis. Based on the clinical information, the spectrum of pathogens detected by the conventional culture method was limited to some species, and the percentage of positive cultures was 91.1% (41/45). In the 45 patients, fifty-two pathogens were identified by culture, 48 (92.3%) of which were accurately identified at the species level (including 40 (76.9%) bacteria, 7 (13.5%) fungi, and 1 (1.9%) *Mycoplasma pneumoniae*, which is considered an atypical pathogen, and the remaining 4 (7.7%) identified at the genus level) (Fig. 1A). The most commonly detected causative pathogens were *Klebsiella pneumoniae* (12/52, 23.1%), *Acinetobacter baumannii* (11/52, 21.2%), *Pseudomonas aeruginosa* (5/52, 9.6%), and *Staphylococcus aureus* (5/52, 9.6%). Other pathogens, such as *Candida albicans* (3/52, 5.8%), *Candida glabrata* (2/52, 3.8%), *Candida krusei* (1/52, 1.9%), *Aspergillus fumigatus* (1/52, 1.9%), and *Mycoplasma pneumoniae* (1/52, 1.9%), were also detected via culture methods. In addition, 7 of 45 (15.56%) patients were coinfected with different kinds of pathogens, including 3 patients with mixed bacteria and fungi, 2 patients with mixed bacteria, and 2 patients with mixed fungi only. The clinical records also revealed that 6 pathogens exhibited antimicrobial resistance. Two of them were recorded as carbapenem-resistant *Klebsiella pneumoniae* and *Acinetobacter baumannii*, respectively, and the other four cases included one resistant strain of *Escherichia coli*, two strains of *Acinetobacter baumannii*, and one resistant strain of *Klebsiella pneumoniae*, none of which reported resistance genes. Detailed information on the pathogens detected by the conventional culture method is shown in Fig. 1 and Table S2.

### Performance of novel POCTs and conventional culture methods for identifying pathogens

A total of 42 samples were detected as positive by the PM Easy Lab RP, with a positive rate of 93.3% (42/45). The majority of the samples (16 patients) contained a single pathogen, 13 samples had two pathogens, 9 samples had three pathogens, the remaining 4 samples had more than three pathogens, and most had 6 pathogens (*Staphylococcus aureus*,* Acinetobacter baumannii*,* Pseudomonas aeruginosa*,* Streptococcus pneumoniae*,* Haemophilus influenzae*,* and Klebsiella pneumoniae*). Coinfections (including viruses) were detected in 5 (11.9%) of the positive samples, 4 of which were positive for RNA viruses, and the most frequently detected RNA virus was Respiratory syncytial virus. There were 2 (Patients 2 and 12) and 3 (Patients 21, 22, and 23) patients with only positive cultures and only positive PM Easy Lab results, respectively.

**Fig. 2:**
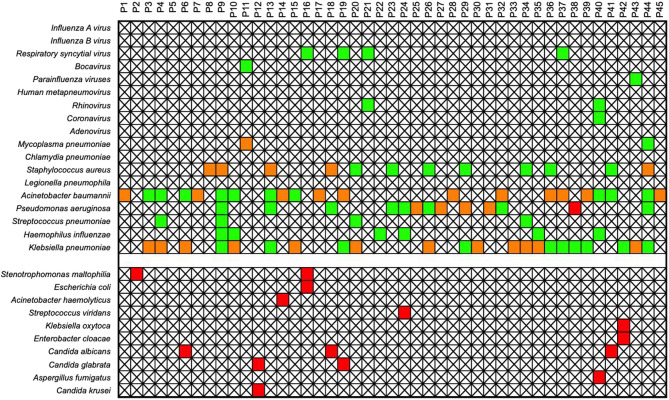
Comparison of pathogen detection between the PM Easy Lab RP panel and conventional culture methods. The culture-positive targets are shown in red, the PM Easy Lab RP-positive targets are shown in green, and the positive targets for both methods are shown in orange. This comparison is restricted to the pathogens targeted by the PM Easy Lab RP assay. The agreement and discrepancies are shown to evaluate the performance of the PM Easy Lab RP against the current clinical standard (culture) for cultivable bacteria and fungi, and to highlight its additional capability in detecting viral targets

Compared with conventional culture methods, the PM Easy Lab can detect more pathogen types, especially viruses, which is predictable, as culture cannot cover viruses. A total of 5 virus types were detected in the 45 samples, including 4 RNA viruses and 1 DNA virus. The PM Easy Lab RP and culture methods were concordant for 36 of 45 (80.0%) patients in the detection range of the PM Easy Lab RP. Among the 36 matched cases, 15 were matched, and the remaining 21 were partially matched, where at least one detected pathogen overlapped between conventional culture and PM Easy Lab RP (Fig. 2, Table S2). Taking culture results as the gold standard, in the detection range of the PM Easy Lab RP, has a sensitivity of 97.1%, a specificity of 27.3%, a positive predictive value (PPV) of 80.5%, and a negative predictive value (NPV) of 75.0%. The overall agreement was 80.0% (Table 2).

**Table 2:** Comparison of positive results and agreement among the PM easy lab and culture methods

Group	Culture positive	Culture negative^a^	Total
PM Easy Lab-positive	33	8	41
PM Easy Lab-negative^b^	1	3	4
Total	34	11	45

^a^Negative: ‘Indeterminate’ culture results and positive cultures out of the range of PM Easy Lab RP

^b^Negative: Mismatched with the culture results within the detection range of the PM Easy Lab RP

### mNGS-based pathogen detection relative to the culture method

**Fig. 3:**
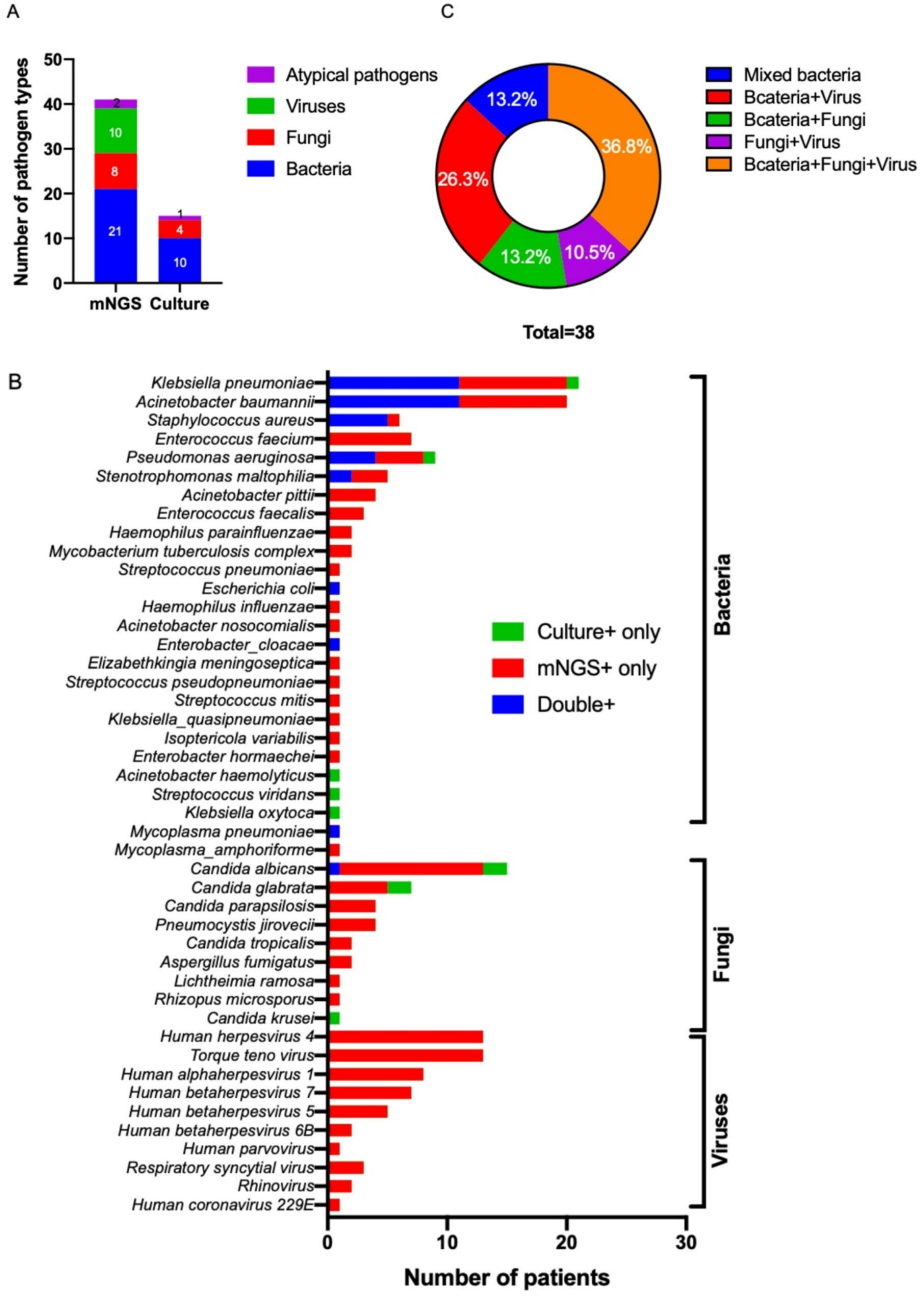
Pathogens identified by mNGS relative to conventional culture methods. **A**-**B** The comparison highlights the broader pathogen spectrum detected by mNGS, particularly for viruses, fastidious organisms, and uncultivable bacteria. **C** The analysis of mixed infections demonstrates the enhanced sensitivity of mNGS in identifying complex polymicrobial infections that are often missed by culture. The culture method serves as a reference for its domain of applicability (cultivable bacteria and fungi)

mNGS DNA detection was performed on samples from all 45 enrolled patients, 8 of whom were simultaneously subjected to both DNA and RNA detection; others were missed by insufficient residual sample volume from these retrospective collections. The detection rates of mNGS for identifying any pathogen and mixed pathogens were 97.8% (44/45) and 84.4% (38/45), respectively. The pathogenic species detected by mNGS consisted of 21 bacteria, 8 fungi, 10 viruses, and 2 *Mycoplasma* strains. The pathogen spectrum revealed that *Klebsiella pneumoniae (20)*,* Acinetobacter baumannii (20)*,* Pseudomonas aeruginosa (8)*,* Enterococcus faecium (7)*,* and Staphylococcus aureus (6)* were the leading bacterial pathogens, whereas *Candida* and Human herpesvirus were the dominant fungal and viral pathogens, respectively, especially *Candida albicans* and Human herpesvirus 4 (EBV). Other pathogens, such as *Mycobacterium tuberculosis complex (2)*, *Pneumocystis jirovecii (4)*,* Mycoplasma pneumoniae (1)*, and RNA viruses (Respiratory syncytial virus, Coronavirus, and Rhinovirus), were also detected. The distribution of pathogens identified by mNGS is shown in Fig. 3A and B.

Inevitably, mNGS detected more pathogen species than culture methods due to its broad-spectrum detection range, especially for viruses and other pathogens that are difficult to culture, such as *Mycobacterium tuberculosis* and *Pneumocystis jirovecii*. As shown in Fig. 3B, for most pathogens, mNGS demonstrated a higher detection rate than culture, not only for viruses but also for *Klebsiella pneumoniae*,* Acinetobacter baumannii*, *Staphylococcus aureus*, *Enterococcus faecium*, and *Candida albicans*. According to the mNGS results, coinfections were detected in 38 patients, and bacteria-fungi-virus and bacteria-virus coinfections were the most common copathogens observed, accounting for 36.8% and 26.3%, respectively (Fig. 3C). The most common pathogens involved in coinfections were *Klebsiella pneumoniae* (19/38, 50.0%) and *Acinetobacter baumannii* (19/38, 50.0%) (Table S2). We also compared the results of mNGS with those of conventional culture methods, and the sensitivity and specificity of mNGS were 90.2% and 25.0%, respectively. The overall consistency rate between culture and mNGS was 82.2%, and the positive predictive value (PPV) of mNGS was 92.5% (Table 3).

**Table 3:** Comparison of positive results and agreement among mNGS and culture methods

Group	Culture positive	Culture negative^a^	Total
mNGS-positive	37	3	40
mNGS-negative	4	1	5
Total	41	4	45

^a^Negative: Contained ‘indeterminate’ culture results

### Comparison between PM easy lab and mNGS

A total of 177 pathogens were detected in the mNGS results, 63 of which were within the target pathogen range of the PM Easy Lab RP assay. Among these 63 isolates, *Acinetobacter baumannii* (20/63, 31.75%), *Klebsiella pneumoniae* (20/63, 31.75%), and *Pseudomonas aeruginosa* (8/63, 12.70%) were the most prevalent, which was consistent with the results of the PM Easy Lab RP. The 114 pathogens found to be out of the detection range by the PM Easy Lab included 15 bacterial species (32/114), 8 fungal species (32/114), 7 viruses (49/114), and 1 *Mycoplasma* species (see Table S2 and S3).

**Fig. 4:**
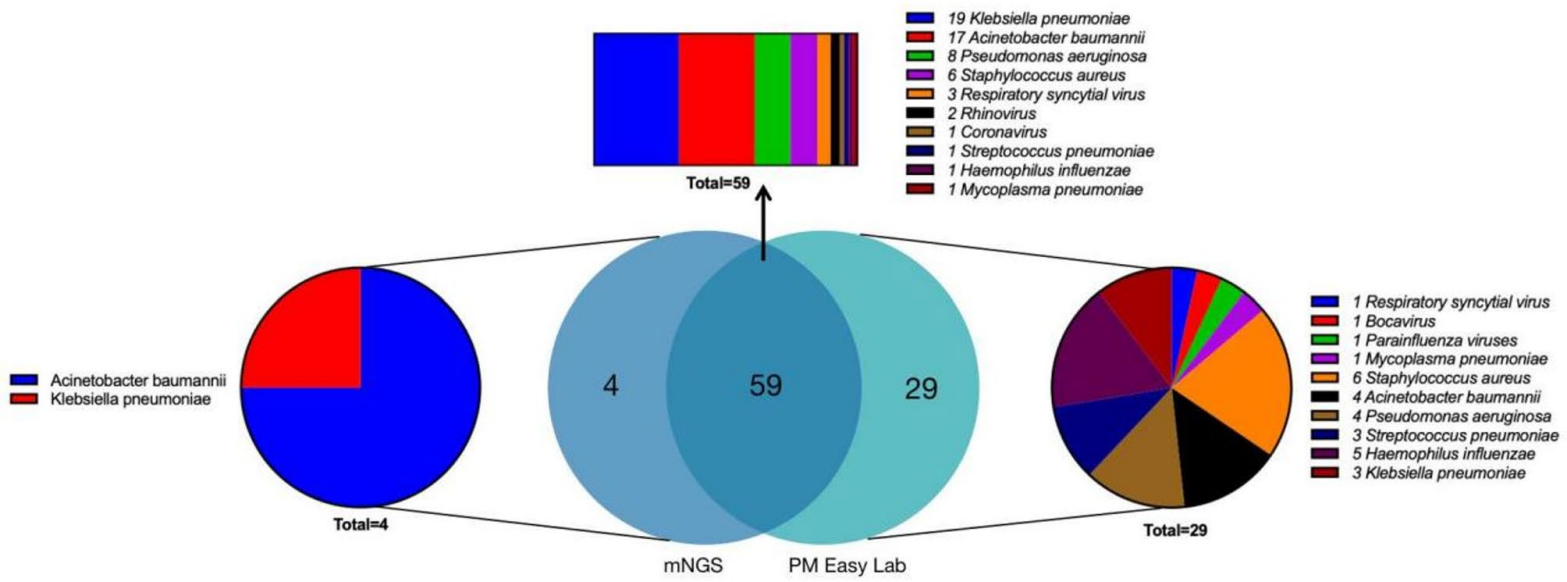
Comparison of the two methods for the identification of pathogens within the detection range of the PM Easy Lab RP

Within the pathogen detection range of the PM Easy Lab assay, 88 pathogens were detected, whereas mNGS detected 63 pathogens. A total of 59 positives were detected by both the PM Easy Lab and mNGS. The 29 discordant positives detected only by the PM Easy Lab assay were *Staphylococcus aureus* (6), *Haemophilus influenzae* (5), *Pseudomonas aeruginosa* (4), *Acinetobacter baumannii* (4), *Klebsiella pneumoniae* (3), *Streptococcus pneumoniae* (3), respiratory syncytial virus (1), Bocavirus (1), Parainfluenza viruses (1), and *Mycoplasma pneumoniae* (1), whereas the 4 discordant positives detected only by mNGS were *Acinetobacter baumannii* (3) and *Klebsiella pneumonia* (1) (Fig. 4).

We also analyzed the results of different methods in terms of patient outcomes. The detection rates of mNGS and PM Easy Lab were comparable (97.8% vs. 93.3%, *P* > 0.05). Furthermore, among the patients who were PM Easy Lab-positive, 37 of 42 had results that were coincident or partly coincident with the mNGS results, while mNGS detected more pathogens due to its wide detection range. In the other 5 patients, mNGS revealed no pathogens within the PM Easy Lab target range, including *Haemophilus influenzae (1)*,* Acinetobacter baumannii (1)*, and *Klebsiella pneumoniae (3).* Among the patients who tested negative by the PM Easy Lab, 2 tested positive by mNGS, and 1 tested negative by both methods. The overall consistency of PM Easy Lab RP and mNGS was 88.9%.

## Discussion

Respiratory tract infections represent a significant public health concern, causing substantial morbidity and mortality, particularly among immunocompromised elderly individuals [1]. The lower respiratory tract can be affected by various pathogens, including bacteria, viruses, fungi, and other microorganisms [15, 16]. Swift and precise identification of these infectious agents is crucial for alleviating patient suffering and preventing antibiotic misuse [17]. Conventional pathogen detection methods, such as staining, microscopic examination, culture, and immunological assays, often yield relatively low detection rates [18, 19]. Recent research has demonstrated that nucleic acid-based detection techniques can significantly enhance the identification of respiratory pathogens. These methods, including multiplex PCR [20–22] and metagenomics [23–25], have shown detection rates ranging from 67.4% to 95.7%, indicating substantial improvement over traditional approaches. Our study demonstrates that both mNGS and PM Easy Lab RP offer significant advantages over conventional culture methods, particularly in detecting mixed infections and viral pathogens. The high positive rates of both molecular methods highlight their potential to address the sensitivity limitations of traditional culture. However, these technologies exhibit fundamentally different operational characteristics that define their distinct clinical roles. The PM Easy Lab RP provides results within 100 min with minimal hands-on time, representing a substantial advantage for rapid point-of-care decision making. In contrast, mNGS requires 24–48 h but offers unbiased detection of a vast spectrum of pathogens.

In this study, the positive rate of the culture method was 91.1%, which was significantly higher than that reported in a previous study [26]. This discrepancy may be attributed to two factors. First, the cases selected for this study required comprehensive clinical records, which often included positive cultures. Second, the selection of the sample type may have influenced the results. The respiratory tract, which is an open environment, is typically contaminated with oral commensal organisms and colonizers [27]. However, mNGS failed to detect culture-positive pathogens, including 1 case of *Pseudomonas aeruginosa* (P38), 1 case of *Klebsiella pneumoniae* (P43), and 1 case of *Viridans streptococcus* (P24). This indicates that the mNGS detection process requires further optimization to increase its sensitivity. The low calculated specificity of mNGS against culture is a known consequence of using a culture-based reference standard, which itself has limited sensitivity. A more clinically relevant interpretation is that the majority of additional pathogens detected by mNGS likely represent true infections, explaining the discordance with culture. This highlights the complementary role of mNGS in identifying fastidious, anaerobic, or antibiotic-affected pathogens that culture frequently misses.

Consistent with previous reports [28, 29], both molecular methods detected high rates of mixed infections. It is important to note that the low rate of mixed infection detection by culture is expected, as this method is not designed to detect viral pathogens, which were commonly identified by the molecular assays. However, the prevalent combinations in our study were bacteria-fungi-virus and bacteria-fungi coinfections, which differ from earlier investigations [30, 31]. This discrepancy may be attributed to variations in sample sizes and mNGS testing conditions across studies.

Our analysis revealed important technical insights. Within its targeted spectrum, PM Easy Lab RP demonstrated higher sensitivity than mNGS for certain pathogens, particularly those with low microbial loads (CT values >35). These samples may indicate relatively low pathogen concentrations that are below the detection limit of mNGS. Furthermore, mNGS failed to detect 2 cases of *Streptococcus pneumoniae* and 2 cases of *Pseudomonas aeruginosa* with CT values less than 35, potentially because of the mNGS pretreatment process. Research has indicated that the host DNA depletion method, which is based on saponin lysis, often results in the loss of *Streptococcus pneumoniae* [14] and *Pseudomonas aeruginosa* [32]. Additionally, mNGS missed two other strains of *Klebsiella pneumoniae* and *Haemophilus influenzae* with CT values of 32.97 and 33.99, respectively, as the detected reads for these pathogens did not meet the reporting threshold. These findings suggest that the criteria for interpreting mNGS detection results require further refinement. However, mNGS detected numerous pathogens beyond the PM Easy Lab RP panel, including fastidious organisms such as *Mycobacterium tuberculosis* and *Pneumocystis jirovecii*, as well as numerous colonizing or opportunistic pathogens capable of causing infection, such as *Candida albicans* and *Haemophilus parainfluenzae*, demonstrating the unique advantages of mNGS in comprehensively detecting pathogens present in samples.

The unbiased nature of mNGS presents novel interpretive challenges. Detection of nucleic acids does not distinguish between active infection, colonization, or environmental contamination, as exemplified by cases of *Pneumocystis jirovecii* and *Candida* detection, which can asymptomatically colonize the respiratory tract. In our study, we also detected 13 cases of *Candida albicans* and 4 cases of *Pneumocystis jirovecii* positive and other pathogens, such as Human herpesvirus and Torque teno virus, which are considered to be associated with patients’ low immunity [33, 34]. For the final diagnosis of responsible pathogens via mNGS, multiple factors need to be considered, including epidemiology, clinical manifestations, imaging results, and outcomes after previous anti-infective treatments. Therefore, molecular findings should be interpreted as highly sensitive but non-specific alerts that require clinical contextualization. The optimal diagnostic approach may involve a hierarchical strategy: initial rapid testing with targeted panels like PM Easy Lab RP, followed by comprehensive mNGS testing in cases of diagnostic uncertainty or treatment failure.

Interestingly, we analyzed the resistance genes of bacteria via mNGS based on the AST results from the clinical records of six samples. The *bla*_*KPC*_ and *bla*_*OXA*_ genes were detected in two samples (P30 and P37), which were consistent with our clinical records. This correlation suggests that mNGS has the potential to provide early insights into antimicrobial resistance, which could be crucial for guiding therapy before traditional susceptibility results are available. However, resistance genes were not detected in the other samples because of the relatively low sequencing depth of the bacteria. Nevertheless, our present study still provides preliminary support for the feasibility of mNGS in drug resistance gene detection. It is important to note that the detection of a resistance gene by mNGS does not definitively confirm its presence in a specific organism without additional validation, as horizontal gene transfer can occur [35].

A pivotal finding is that these technologies should be viewed as complementary rather than competitive. PM Easy Lab RP is ideally suited for frontline settings where rapid detection of common respiratory pathogens, such as *Klebsiella pneumoniae*,* Acinetobacter baumannii*,* Pseudomonas aeruginosa*,* Staphylococcus aureus*, and *Streptococcus pneumoniae*, as reported in previous studies [24, 36, 37], can guide immediate treatment decisions, potentially reducing unnecessary antibiotic use and shortening hospital stays. Its targeted approach offers operational efficiency but limited scope. Conversely, mNGS serves as a powerful tool for complex cases where conventional methods fail, particularly in immunocompromised patients or those with unresolved infections. Its comprehensive detection capability justifies the longer turnaround time and higher cost in these scenarios, potentially preventing prolonged diagnostic odysseys and inappropriate treatments. Future studies should incorporate formal health economic analyses to quantify the cost-benefit ratio of these technologies when used individually or within integrated diagnostic pathways. However, a fundamental distinction from culture-based methods is their inability to confirm microbial viability. The detection of pathogen-derived DNA/RNA could represent active infection, historical infection with residual nucleic acid, or mere colonization. Thus, culture, despite its lower sensitivity and longer turnaround time, retains the critical advantage of confirming the presence of viable organisms, which is indispensable for obtaining antimicrobial susceptibility testing (AST) to guide targeted therapy.

Our study has several limitations that should be acknowledged. First, its retrospective nature and relatively small sample size may limit the generalizability of our findings and preclude formal cost-effectiveness analysis. Second, RNA sequencing was only performed on a subset of samples (*n* = 8/45) due to insufficient residual sample volume, potentially leading to underdetection of RNA viruses and an underestimation of virus-related mixed infections. Third, the molecular tests were conducted in a centralized third-party laboratory, while culture methods were performed in various hospital settings; differences in testing conditions and protocols may have influenced concordance. Finally, the lack of clinical outcome data prevents correlating diagnostic findings with treatment efficacy or patient prognosis. Future prospective studies with larger cohorts and standardized testing conditions are warranted to validate these observations.

## Conclusions

In conclusion, both mNGS and PM Easy Lab RP represent significant advances in diagnostic microbiology for pulmonary infections. PM Easy Lab RP offers speed and efficiency ideal for frontline use, while mNGS provides comprehensive detection valuable for complex cases. Understanding their complementary strengths and limitations will enable clinicians to deploy these technologies effectively within hierarchical diagnostic pathways, ultimately contributing to more precise and timely patient management.

## Supplementary Information

Supplementary Material 1.

Supplementary Material 2.

Supplementary Material 3.

## Data Availability

The data reported in this study are also available in the CNGB Nucleotide Sequence Archive (CNSA: https://db.cngb.org/cnsa; accession number CNP0004100).

## Abbreviations

POCT: Point-of-care testing
mNGS: Metagenomic next-generation sequencing
RP: Respiratory Panel
PPV: Positive predictive value
NPV: Negative predictive value
BALF: Bronchoalveolar lavage fluid
AST: Antimicrobial susceptibility test
CCCLS: Chinese Committee for Clinical Laboratory Standards
DNBs: DNA nanoballs
RCA: Rolling circle amplification
BWA: Burrows‒Wheeler alignment
SMRN: Strictly mapped reads
IQRs: Interquartile ranges
CIs: Confidence intervals
CRP: C-reactive protein
PCT: Procalcitonin
CT: Cycle threshold
